# Western gray whale behavioral response to seismic surveys during their foraging season

**DOI:** 10.1007/s10661-022-10023-w

**Published:** 2022-10-18

**Authors:** Glenn Gailey, Olga Sychenko, Mikhail Zykov, Alexander Rutenko, Arny Blanchard, Rodger H. Melton

**Affiliations:** 1 Cetacean EcoSystem Research, Olympia, WA USA; 2 JASCO Applied Sciences (Canada) Ltd, Victoria, BC Canada; 3 V.I. Il’ichev Pacific Oceanological Institute FEB RAS, Vladivostok, Russia; 4 Blanchard Ecological, Fairbanks, AK USA; 5 ExxonMobil Exploration Company, Spring, TX USA

**Keywords:** Western gray whale, Seismic, Movement, Respiration, Behavioral response, Vessel separation, Sound exposure levels

## Abstract

Gray whales utilizing their foraging grounds off northeastern Sakhalin Island, Russia, have been increasingly exposed to anthropogenic activities related to oil and gas development over the past two decades. In 2015, four seismic vessels, contracted by two operators, conducted surveys near and within the gray whale feeding grounds. Mitigation and monitoring plans were developed prior to the survey and implemented in the field, with real-time data transfers to assist the implementation of measures aimed at minimizing impacts of acoustic exposure. This study examined the behavioral response of gray whales relative to vessel proximities and sounds generated during seismic exploration. Five shore-based teams monitored gray whale behavior from 1 June to 30 September using theodolite tracking and focal follow methodologies. Behavioral data were combined with acoustic and benthic information from studies conducted during the same period. A total of 1270 tracks (mean duration = 0.9 h) and 401 focal follows (1.1 h) were collected with gray whales exposed to sounds ranging from 59 to 172 dB re 1 μPa^2^ SPL. Mixed models were used to examine 13 movement and 10 respiration response variables relative to “natural,” acoustic, and non-acoustic explanatory variables. Water depth and behavioral state were the largest predictors of gray whale movement and respiration patterns. As vessels approached whales with increasing seismic/vessel sound exposure levels and decreasing distances, several gray whale movement and respiration response variables significantly changed (increasing speed, directionality, surface time, respiration intervals, etc.). Although the mitigation measures employed could have reduced larger/long-term responses and sensitization to the seismic activities, this study illustrates that mitigation measures did not eliminate behavioral responses, at least in the short-term, of feeding gray whales to the activities.

## Introduction

Gray whales (*Eschrichtius robustus*) have been described as both ecosystem sentinels and engineers (Moore, 2008; Moore & Reeves, 2018; Nelson & Johnson, 1987). Their benthic foraging strategy makes them unique among cetaceans, particularly among baleen whale species. Their mode of feeding can re-suspend large amounts of nutrients and sediments into the environment that can be equated to geological processes (Nelson & Johnson, 1987). Globally, gray whales exist in the northern hemisphere of the Pacific Ocean. Historically, gray whales were present in the northern hemisphere of the Atlantic Ocean, but whaling in the seventeenth century drove the Atlantic population into extinction (Mead & Mitchell, 1984). The Pacific eastern gray whale population also suffered severely from whaling primarily due to their coastal affinity and concentration on their breeding and foraging habitats. After post-modern whaling, eastern gray whales were listed as endangered by the International Union for Conservation of Nature (IUCN). Due to a number of conservation measures, eastern gray whales rebounded to pre-whaling numbers in what amounts to one of the most successful conservation stories today (Jones & Swartz, 2009; Reilly et al., 1983; Rugh et al., 2005).

On the western side of the Pacific Ocean, another population of gray whales, the western gray whale, was previously thought to have been extinct due to whaling (Bowen, 1974; Brownell & Chun, 1977). However, studies in Russia discovered a remnant and genetically distinct population foraging in a relatively small spatial area off northeastern Sakhalin Island, Russia (Lang et al., 2011; LeDuc et al., 2002; Weller et al., 2002a). After years of research in the late 1990s to obtain sufficient population and genetic data, studies indicated the population was small (< 150 individuals) with fewer than 25 reproductive females (Weller et al., 2002a). Based on these data, the IUCN initially classified the western gray whale population as critically endangered. More recently, based on population growth rates and inclusion of data from Sakhalin and Kamchatka, Russia, IUCN changed the population status to endangered (Cooke et al., 2018). Cooke et al. (2018) noted, however, that the population would still be considered critically endangered based on individuals that were only observed off Sakhalin due to the low number of reproductive females.

One of the major concerns towards the future survival of the population was the discovery of large oil and gas reserves that existed in close proximity to the western gray whales’ feeding grounds. Since the mid-1990s, the level of anthropogenic pressure on the Sakhalin gray whale feeding grounds has increased; what was once a relatively pristine area has been subjected to an ever-growing amount of vessel traffic, dredging, pipeline placement, platform installations, pile driving, seismic exploration activity, and, more recently, large-scale salmon fishery operations (Gailey, 2013; Gailey et al., 2007, 2016, 2022; Lowry et al., 2018; Muir et al., 2015a, b, 2016; Yazvenko et al., 2007). The amount of activity within a single summer foraging period culminated in 2015 with two operators conducting four seismic surveys that spanned the majority of the gray whale foraging season and habitat off Sakhalin Island.

When the oil and gas industry began working off Sakhalin in the late 1990s, little was known about gray whale responses to anthropogenic activities. A few studies have suggested gray whales abandoned entire breeding lagoons in response to increasing vessel activity (Bryant et al., 1984; Gard, 1978). Behavioral response studies on the eastern gray whale feeding grounds suggested that 10% of gray whales would respond to received root mean square seismic sound pressure levels (SPL) above 163 dB re 1 μPa^2^ (Malme et al., 1986, 1988). Behavioral response studies on western gray whales found that gray whales significantly changed their movement, respiration, abundance, and distribution despite employing mitigation approaches to minimize acoustic exposure levels (Gailey et al., 2007; Weller et al., 2002b; Yazvenko et al., 2007). More recent impact studies on gray whale distribution and behavior during a seismic survey in 2010, however, found little to no responses to the activity (Gailey et al., 2016; Muir et al., 2015a, b, 2016). It remained unclear if gray whales tolerated or habituated to the activities or if, as suggested by the authors, the impact studies were too limited in sample sizes to detect responses given the small number of whales observed and the short duration (3 weeks) of the survey. In fact, Gailey et al. (2016) provided power analyses of their dataset that documented their inability to detect subtle to moderate levels of potential changes in movement and respiration given their sample sizes.

Due to the potential sensitivities to the presence of anthropogenic activity in close proximity to the western gray whale feeding grounds, two oil and gas companies undertaking seismic operations (Exxon Neftegas Limited (ENL) and Sakhalin Energy Investment Company (SEIC) developed mitigation measures to limit sound levels received by gray whales to less than 163 dB re 1 μPa^2^ SPL. Comprehensive monitoring programs were developed to better understand the gray whale population, their habitat, and the potential impacts of industrial activities as well as to examine the effectiveness of the applied mitigation strategies (Bröker et al., 2015; Johnson et al., 2007).

In 2015, both SEIC and ENL conducted seismic surveys within four of their licensed blocks. The surveys involved up to four seismic source vessels that spanned both the duration and spatial extent of the western gray whale feeding season and habitat. The number of seismic vessels and areas of exploration varied throughout the feeding season. In addition, mitigation measures taken by both companies deviated from previous mitigation strategies by not actively implementing the 163 dB re 1μPa^2^ airgun shutdowns aimed at reducing behavioral disturbance of gray whales during the early part of their respective surveys, when few whales would be present, to facilitate completion of activities as soon as possible.

Later in the season, airgun shutdowns were implemented to avoid exposure of animals to sound levels > 163 dB re 1μPa^2^ SPL, but the two companies applied this mitigation measure differently, with ENL employing behavioral mitigation criteria for all individuals while SEIC restricted the criteria to only mother-calf pairs (Aerts et al., 2022; Rutenko et al., 2022; SEIC, 2015). This was arguably a less stringent approach than previous mitigation measures developed for western gray whales (Bröker et al., 2015; Johnson et al., 2007). The monitoring studies undertaken in 2015 did provide an opportunity to collect sufficient samples and to observe gray whales being exposed to higher sound levels than during previous impact studies. As such, one operator (ENL) implemented a comprehensive and extensive monitoring program (Aerts et al., 2022). The research design monitored not only ENL but SEIC activities as well.

In this study, we examine if western gray whale movement and respiration patterns were influenced by sound (seismic, onshore pile driving, and vessel sound) and/or proximity of the activities that occurred. We sought to include environmental, temporal, and spatial related variables that have previously been shown to account for variability in gray whale movement and respiration patterns (Gailey, 2013; Gailey et al., 2007, 2016). One crucial consideration that was not considered or available in previous studies was prey availability, and we provide an initial examination of these data in this study.

## Methods

### Study site, observation platforms, and seismic surveys

**Fig. 1:**
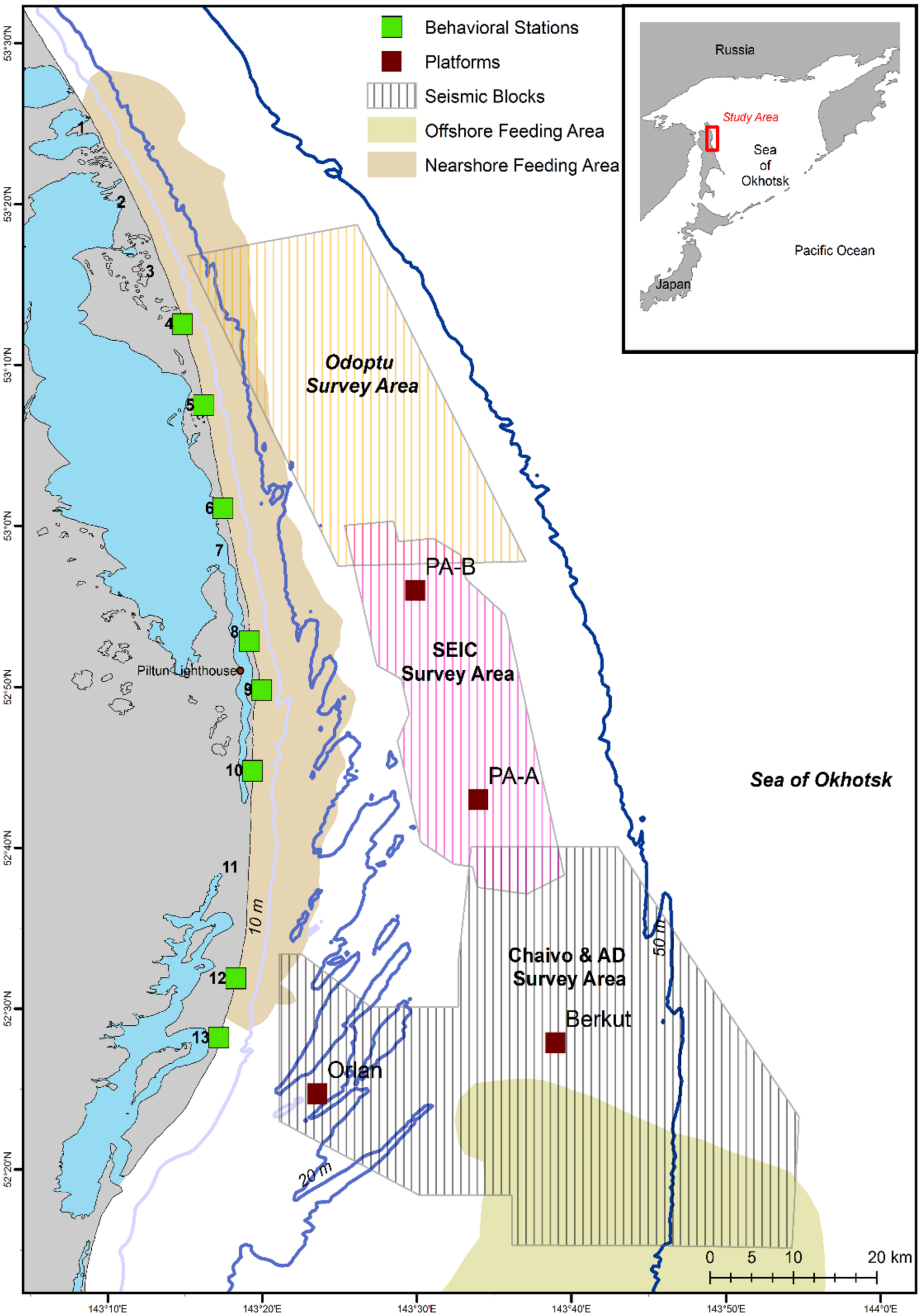
Map of the study area illustrating the known nearshore and offshore western gray whale feeding areas and four seismic survey areas where exploration activity was conducted in 2015

During the summer of 2015, a total of four seismic source vessels operated off northeastern Sakhalin Island, Russia, to capture data about oil and gas reservoirs within four license blocks near or within the western gray whale feeding grounds (Fig. 1). The seismic operations were spatially and temporally coordinated by the two operators with seismic lines closest to the feeding areas being acquired as early in the feeding season as possible. Aerts et al. (2022) summarizes the seismic operations as well as the mitigation and monitoring approaches taken in 2015.

Five shore-based behavioral teams monitored the movements, behavior state, and respiration patterns of western gray whales from eight separate locations during the seismic operations. The locations of behavioral observations were dependent on the seismic survey activity that occurred at the time. Two teams observed gray whales from stations 4 and 6 throughout the study period (1 June–30 Sept). The three other teams occupied stations 5, 12, and 13 before and during the Odoptu and Chaivo seismic surveys, but moved to stations 8, 9, and 10 when the Piltun-Astokh seismic survey started and remained there until 30 Sept (Fig. 1). Due to the relatively low elevations of the onshore stations, wooden towers (4-m height) were custom built at each station, with the exception of station 6, to increase the observation range of the stations. The towers had an independent structure in the center to provide a stable platform for the theodolite. Observation height from the stations ranged from 10.8 to 23.7 m. These elevations were considered to be sufficiently high to monitor the nearshore feeding ground of the western gray whales that generally extends less than 15 km from shore with gray whales occurring on average approximately 1.5 km from shore (Gailey, 2013; Gailey et al., 2016; Muir et al., 2015a, b, 2016) (Fig. 1).

### Movement and respiration data collection

Gray whales were monitored from five of the eight stations during each good-weather day (good visibility with Beaufort Sea State < 5). An individual or group of gray whales was tracked from shore using theodolite tracking techniques (Gailey & Ortega-Ortiz, 2002; Würsig et al., 1991). At each station, a Sokkia DT5A or DT540 theodolite was used that had a 30 × monocular magnification and 5 arc-sec level of angular precision and tilt compensation. The maximum distance from a station that a gray whale was tracked depended on the observation height of that station. At lower elevation stations, gray whales were tracked up to approximately 6 km from the station, while at higher elevation stations, whales were tracked to distances up to 12 km. Whales were continuously tracked until the whales were either no longer visible or environmental conditions hampered reliable tracking. Gray whales observed closest to either the nearest vessel or seismic activity were preferentially tracked throughout the study with single or recognizable individuals being favored to avoid measurement errors within the track. The geographic positions of the whales were estimated in real-time and visually displayed in a custom version of the Pythagoras theodolite data collection system (Gailey & Ortega-Ortiz, 2002).

Focal follow observation techniques were used to record respiration patterns of gray whales in the field (Altmann, 1974; Martin et al., 1993). Focal follows were conducted only on single or individual recognizable whales to ensure respiration events were not missed during data collection. A focal follow included at least one observer visually focusing on the whale’s location aided by 7 × 50 Fujinon FMTRC-SX binoculars. The focal observer verbally stated a behavioral event (respiration, peduncle arch, etc.), which was immediately recorded by a computer operator using a programmable keyboard connected to the Pythagoras behavioral software (Gailey & Ortega-Ortiz, 2002). Focal follow sessions were recorded in conjunction with theodolite tracking of the same whale, which provided the ability to link respiration information to the spatial location of the animal. A focal follow session ceased if the whale moved out of the observation area or environmental conditions were unacceptable (visibility < 5 km, Beaufort Sea State > 3 or wind gust speeds > 20 km h^−1^).

### Behavioral response and explanatory variables

Definitions of all response and explanatory variables are provided in Table 1 of Gailey et al. (2016). Data processing and response variables were consistent with previous behavioral studies of western gray whales (Gailey, 2013; Gailey et al., 2007, 2016). All movement data derived from theodolite tracking were resampled based on a 90-s criterion to avoid over- or under-sampling issues and to standardize step lengths (Turchin, 1998). For every resampled track, movement response variables (whale speed, directionality, reorientation rate, etc.) were calculated at 10.5-min intervals (hereafter referred to as a “bin”). This bin duration was chosen to examine short-term changes in movement patterns. Observations of gray whale respirations at the surface during focal follows were processed to measure the animal’s respiration interval, dive time, surface time, number of blows per surfacing, percent time at surface, surface blow rate, and dive surface blow rate. These respiration variables were also averaged over 10.5 min to be consistent with the movement variables.

To account for parameters that may alter gray whale movement and respiration patterns, we included two types of explanatory variables: natural and impact variables. In this study, we selectively chose only those natural variables that in previous analyses explained a significant amount of variation in gray whale movement and respiration response variables (Gailey, 2013; Gailey et al., 2007, 2016). This simplified the behavior response models by reducing the number of variables considered. Natural explanatory variables considered in the behavioral response models were the observation station, date of the observation, time of day, Beaufort Sea State, visibility, water depth, tide height, and behavioral state. Behavioral state was classified as feeding, feeding/traveling, traveling, or mixed. Mixed behavior denoted any combination of unknown, transitional, or unrecognized behavior as well as other seldom observed behavioral states, such as resting, milling, and social.

Anthropogenic variables (e.g., “impact” explanatory variables) that were hypothesized to change gray whale behavior, such as sound exposure and/or vessel distance, were also included in the analyses. Acoustic monitoring data (Rutenko et al., 2022) were used to calculate received levels for several acoustic metrics, which included (1) sound exposure level (SEL), (2) RMS sound pressure level (SPL), and (3) peak sound pressure level (PK). All acoustic metrics were estimated for pulse (pile driving/seismic) sounds, while only SEL and SPL were estimated for continuous (vessel) sounds in the study area. Two explanatory variables based on the SEL metric were computed for every 30-s increment along a whale track: the SEL over the current time step (SEL_30s_) and the SEL accumulated from the beginning of the track to the current time step (cSEL). Vessel activity was recorded in the field using a combination of four separate automatic identification system (AIS) receivers, satellite AIS, and vessel GPS logs. Non-acoustic variables considered in the response models were the closest vessel approach (km), number of vessels, and the relative orientation of the vessel to the whale (ROW).

### Acoustic monitoring and sound level estimation

Vessel, pile driving, and seismic survey sounds were recorded with 40 automatic underwater acoustic recorders (AUARs) with frequency range of 2 to 15,000 Hz. Nine of these AUARs (denoted as RI-AUARs) were equipped with both an iridium and a digital radio-telemetric channel that provided real-time acoustic waveform data within the 2- to 2000-Hz frequency band with a potential dynamic range of 145 dB (Rutenko et al., 2022). The RI-AUARs were equally spaced along the offshore boundary of the gray whale feeding area (Fig. 1) and provided real-time data to a shore-based acoustic monitoring team. Thirty-one monitoring sites equipped with non-telemetric AUARs were located closer to shore in water depths of 10 m as well as in deeper waters to provide detailed information on sound propagation for post hoc analyses. Rutenko et al. (2022) provide further details of acoustic data acquisition during the seismic surveys.

### Prey availability

As recommended by Gailey et al. (2007, 2016), prey biomass and availability could be important variables to consider while examining gray whale movement and respiration patterns. We explored the maximum prey biomass observed at the animal’s location over the course of each bin. Blanchard et al. (2022a, b) provides a summary of the benthic prey biomass data and data collection methods used in 2015. Besides repeat sampling of the 2002–2014 benthic grid covering the Sakhalin gray whale feeding areas described in Blanchard et al. (2022a, b), the 2015 benthic study mainly focused on examining the spatial and within season changes of prey availability. A finer scale sampling grid, covering waters of < 20 m within the nearshore feeding area, was sampled three different times within the gray whale foraging season (Blanchard et al., 2022a, b). Interpolation methods were required to estimate the biomass of each group at the gray whale location. To estimate benthic biomass at the gray whale location, we applied both kriging and inverse distance weighted (IDW) interpolation approaches. The kriging approach considered spatial variables and water depth in the interpolation, while IDW simply uses distance to other neighboring data points. Behavior and prey analyses were conducted separately from the behavioral models examining potential disturbances to whales, since the interpolated area of prey availability did not cover whales observed outside the 20-m isobath. The prey groups that were examined here were (1) amphipods, (2) isopods, (3) Cumaceans, (4) Polychaetes, (5) bivalves, and (6) total biomass (summation of groups 1–5). ANOVAs were used to examine differences in prey biomass at the whales’ locations among the different behavioral states.

### Behavioral response models

The behavioral response models were used to determine associations of the response variables (e.g., whale speed, dive time) to the natural (e.g., water depth, time of day, behavioral state) and impact (e.g., SEL_30_, closest vessel distance) explanatory variables. The model approach assumed the response variables had an approximately normal distribution. To meet this assumption, transformation methods were applied. Logit transformations were applied to linearity and mean direction indices, while natural log transformations were applied to speed, distance from shore, and respiration variables. Collinearity among covariates was examined using pair-wise Pearson correlation coefficients for all continuous variables, and box-plots were used to assess non-continuous against continuous variables. A correlation coefficient larger than 0.60 warranted concern that one covariate could mask the effects of another.

Associations among the variables of interest were examined using mixed linear models (Pinheiro & Bates, 2000). This modeling approach was chosen because autocorrelation was potentially present due to the time series nature of the sequential bins of observation. Autocorrelation within tracks was accounted for by estimating mixed linear models that assumed unstructured, constant, or autoregressive dependencies in model residuals. Another analytical challenge was the potential issue of pseudo-replication with a single track or focal follow having more or less representative bins within the dataset compared to others. To adjust for this bias, we weighted each observation by a value inversely proportional to the probability of obtaining that observation (Horvitz & Thompson, 1952; Overton & Stehman, 1995). Model effects were estimated with generalized estimating equations using the R function “lme” available in the “nlme” package (Pinheiro et al., 2018). Model selection was based on a stepwise selection procedure that relied on the Bayesian information criterion (BIC). BIC was chosen as the measure of variable utility because it generally yields a more parsimonious model. Both forward and backward step selections were used to include natural and/or impact effects. Standardized residual plots were inspected to assess model fit.

## Results

### Effort

Seismic surveys by the two operators were carried out from 11 Jun to 23 Sept 2015. The Odoptu survey was acquired from 11 Jun to 7 July, the Piltun-Astokh survey from 8 to 25 Jul, and the Chaivo/Arkutun-Dagi survey from 7 Jul to 23 Sept (see Aerts et al., 2022) for further details about the seismic operations). The movement and respiration patterns of gray whales were monitored from 1 Jun to 30 Sept 2015 by five shore-based behavior teams (Fig. 1). The total number of observation days at each station ranged from 24 to 85 days with an average of 480 h (cumulatively 3843 h) of effort being conducted among the behavioral teams. Station 5 had the least amount of effort. The mean number of observation days per team was 88 (range 70–101). A total of 1270 Gy whale tracks and 401 focal follows were collected during the field season. The average duration of a gray whale track was 0.9 h (range = 0.2–13.1 h) with a total of 44,634 geographic positions of gray whales recorded. The mean focal follow session lasted 1.1 h (0.2–12.2 h) with 30,735 respiration events collected. A total of 7403 movement and 2328 respiration bins were derived for the analyses. Gray whales were observed feeding in a localized area 26% of the time, while feeding/traveling (or searching) behavior was observed 33% of the time. Approximately 30% of the gray whale activity budget in 2015 was spent on traveling, while the remaining 11% of the behaviors observed were a mixture of milling, socializing, resting, nursing, etc. The combined foraging behavior (59%) was similar to those previously reported for western gray whales (Gailey, 2013; Sychenko, 2011; Villegas-Amtmann et al., 2015), but slightly less time was spent on concentrated feeding compared to the observed feeding/traveling behaviors.

### Response variables

Gray whale movement and respiration response variables were highly associated with one another and with behavioral states (feeding, traveling, etc.). Traveling gray whales exhibited higher speeds with increased spatial range and more directional movement than non-traveling gray whales, while feeding gray whales’ reorientation rate was more variable with less geographical movement than non-feeding gray whales. While gray whales were feeding, they typically increased their dive time, decreased the interval between subsequent respirations, shortened their surface time, and had a higher surface blow rate (number of blows per surface time). In general, gray whales were minimizing their surface time and maximizing their time underwater while engaged in feeding compared to traveling.

**Fig. 2:**
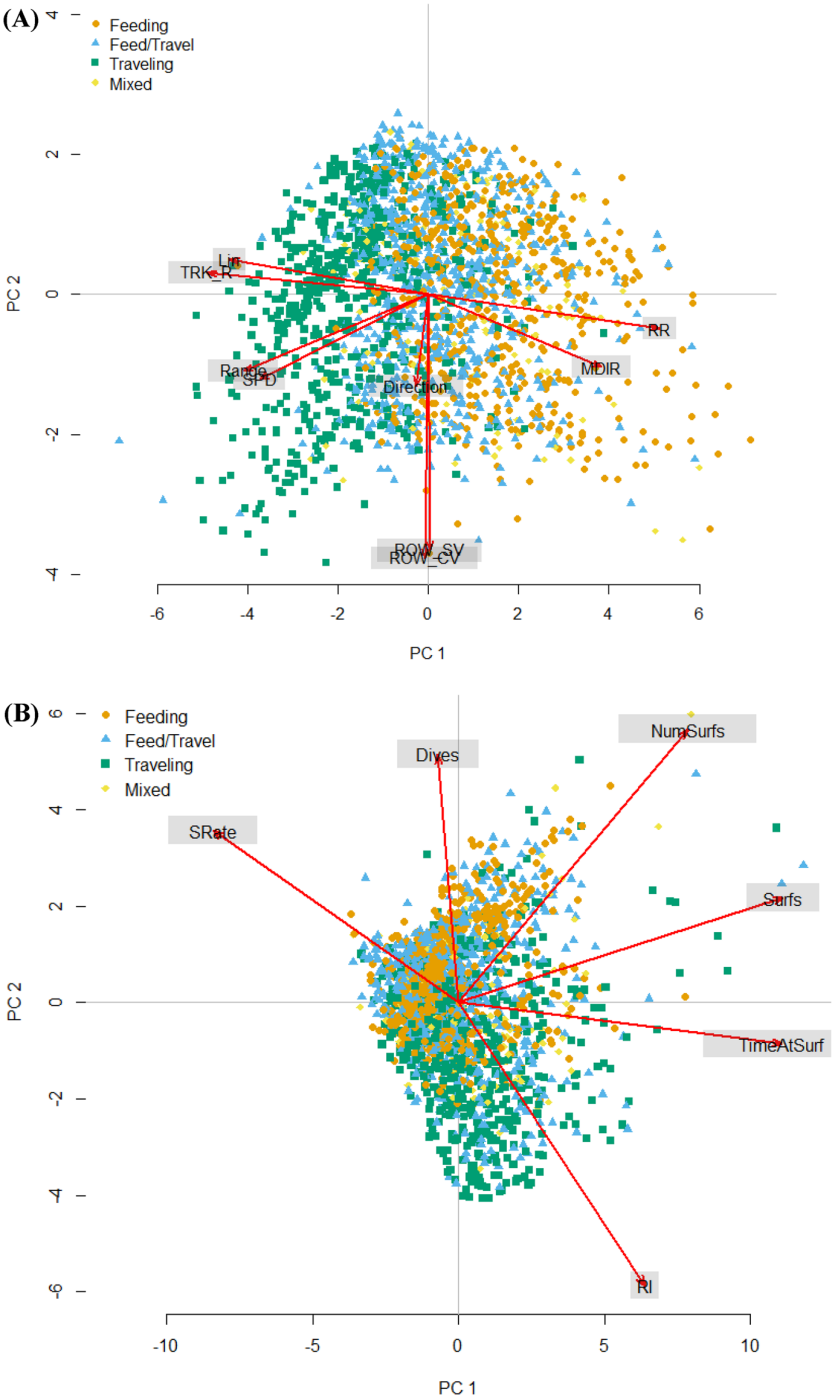
Principle component analysis (PCA) of western gray whale movement (**A**) and respiration (**B**) response variables. Movement variables were defined as SPD (speed), Range (range index), TRK_R (directionality index), LIN (linearity), RR (reorientation rate), MDIR (mean direction), ROW_SV (relative orientation to closest seismic vessel), ROW_CV (relative orientation to closest vessel), and Direction (spatial direction of movement). Respiration variables were defined as SRate (surface blow rate), Dives (dive time), NumSurfs (number of blows per surfacing), Surfs (surface time), TimeAtSurface (percent time at surface), and RI (respiration interval)

Principal component analyses (PCA) were applied to the response variables of movement and respiration to examine if a combination of response variables (speed, linearity, reorientation rate, etc.) would be a better indicator of response as opposed to examining each response variable separately. There are a number of inter-relationships among the response variables. For example, traveling animals tend to do so more linearly at higher speeds and increased range as opposed to foraging animals. When gray whales are feeding, they tend to increase their dive time and decrease the time at the surface with shorter respiration intervals. The principal component scores (e.g., PC1, PC2, PC3) were used as response variables in separate models. The first three component scores for movement (69% of the variation) and respiration (92% of the variation) were used in the analyses (Fig. 2). The first two components were interpreted to explain variation in the relationships of the different movement and respiration among the different behavioral states, while the third component was interpreted to account for the remaining variation that could be related to other natural or impact related variables.

### Acoustic exposure

**Fig. 3:**
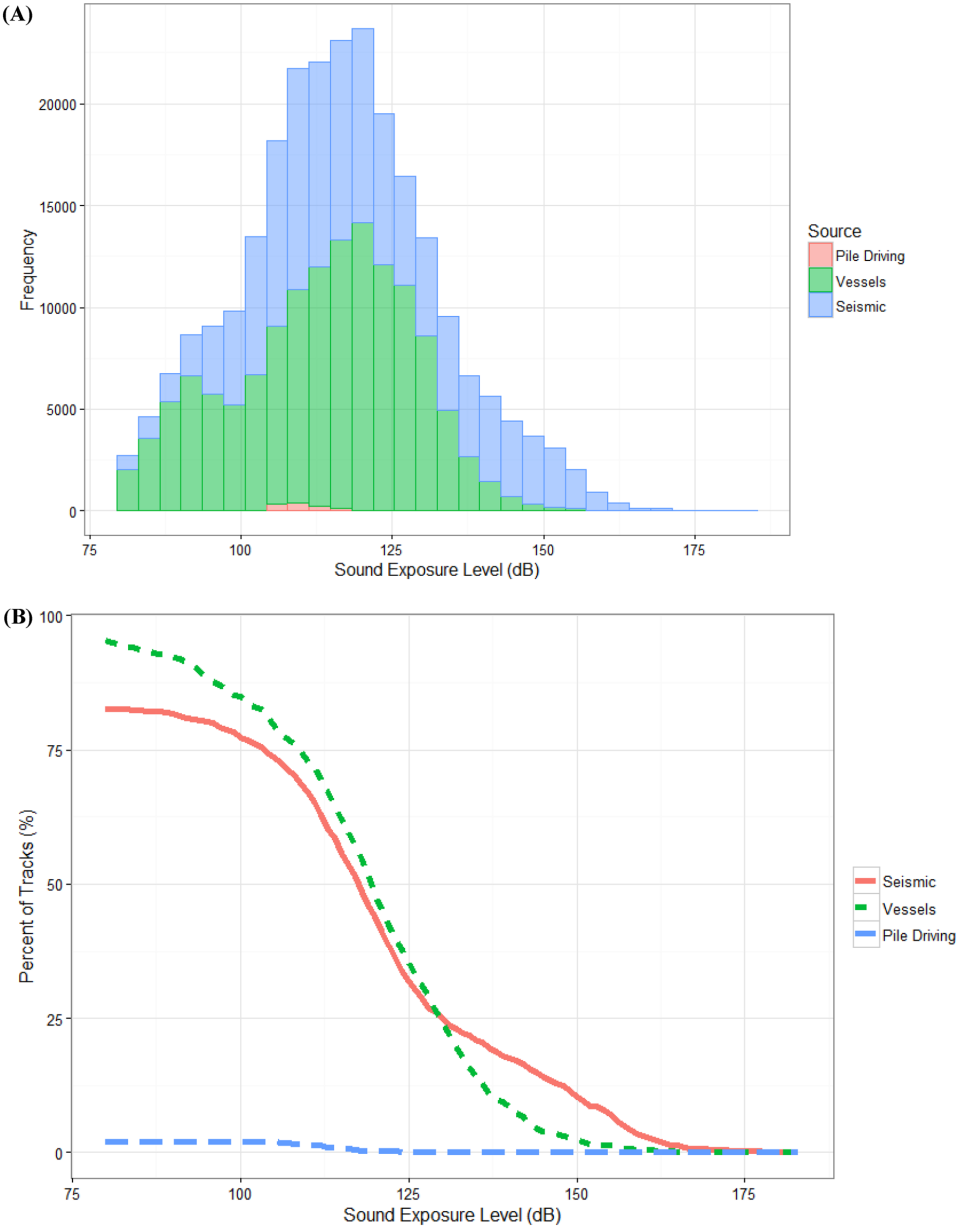
**A** Histogram of acoustic exposure (SEL_30s_) for all the movement data and **B** proportion of the tracks receiving acoustic exposure from seismic, vessel, and pile driving activities

Western gray whales were exposed to pile driving, seismic, and vessel sounds in 2015 with a few whales (< 1.2% of the dataset) exposed to all three at the same time. The onshore pile driving activity was spatially localized and of short duration. Gray whale exposure to pile driving sounds occurred on 14 different days with a mean SEL_30s_ of 110 dB re 1 μPa^2^s (range 97–123). Vessel activity occurred throughout the foraging season but varied spatially and in intensity. Gray whale exposure to vessel sounds had a mean SEL_30s_ of 113 dB re 1 μPa^2^s (59–156). Exposure to vessel sounds was observed in 68% of the behavioral dataset in this study. Gray whale exposure to seismic pulses had a mean SEL_30s_ of 120 dB re 1 μPa^2^s (53–172). Over 55% of the behavioral dataset showed exposure to some degree of seismic sounds. However, gray whale exposure to particularly high sound levels (SEL_30s_ > 156 dB re 1 μPa^2^s) for vessel or seismic activity were infrequent (Fig. 3). Due to high collinearity (Pearson’s correlation > 0.95) among the acoustic metrics, only SEL_30s_ and cSEL were included in the behavioral response models. Due to the low levels and small sample size of pile driving sounds, pile driving was not further considered in the behavioral response models.

### Prey availability

The median prey biomass values at gray whale locations from the kriging and IDW interpolations varied (Table 1), with IDW approach generally yielding higher values. Amphipod biomass was significantly lower while feeding/traveling (df = 3, *F* = 9.88, *P* < 0.001) for kriging, while biomasses were significantly higher during feeding and feeding/traveling (3, 11.46, < 0.001) compared to traveling or mixed behaviors. A similar pattern was observed for isopods for kriging (3, 9.06, < 0.001) and IDW (3, 7.45, < 0.001) approaches. Bivalve median biomasses were observed to be significantly lower during mixed behaviors for kriging (3, 35.93, < 0.001), but higher during traveling (3, 22.47, < 0.001) with IDW. Polychaete and cumacean median biomass were slightly lower during mixed behaviors for kriging and IDW. Total biomass was lowest, while animals were feeding with the kriging (3, 50.00, < 0.001) interpolation but slightly higher with IDW (3, 12.34, < 0.001).

**Table 1:** Median biomass (g/m^2^) estimated from two interpolation approaches at western gray whale locations while engaged in different behaviors

		Amphipods	Isopods	Bivalves	Polychaetes	Cumaceans	Total biomass
Kriging	Traveling	37.8	6.9	12.5	2.0	0.5	353.9
	Feed/Travel	34.8	10.0	13.3	2.3	0.5	328.6
	Feeding	38.3	6.5	11.5	2.2	0.5	324.4
	Mixed	39.6	6.0	3.3	1.6	0.3	369.1
IDW	Traveling	39.0	8.4	36.1	3.3	0.6	394.5
	Feed/Travel	44.3	11.3	24.8	2.9	0.5	398.0
	Feeding	43.4	10.2	26.8	3.5	0.7	400.2
	Mixed	38.9	9.9	25.5	2.9	0.5	395.0

Inclusion of prey biomass in the behavioral response models yielded mixed results. For example, amphipod significantly entered into the dive time model for kriging but not for IDW. As the duration of the dive time significantly increases with water depth (below) and the kriging interpolation used water depth to adjust the biomass, it is likely that these results reflected covariance with water depth. By including behavioral state in the movement and respiration models, it largely accounted for differences while engaged in foraging compared to prey types and biomass values. In other words, while engaged in feeding behaviors, gray whales were not demonstrably responding by moving slower, changing directions more, or breathing quicker where biomass was higher or in association with prey type. For this reason and due to the number of extraneous variables, we excluded prey biomass as a potential explanatory variable in the behavioral response models.

#### Behavioral response to anthropogenic sound and proximity

The behavioral state and water depth were the largest predictors of the respiration response variables. As gray whales observed off Sakhalin feed, they decrease their respiration interval and time at the surface, but increase their dive time, number of blows per surfacing, and surface blow rate. When gray whales were observed in deeper waters, they slightly increased their respiration interval, surface time, blows per surfacing, and dive time. There was also a decrease in the surface blow rate and dive-surface blow rate associated with increased water depth. Tide height entered into several of the respiration response models but was non-significant. With increased tide height, the surface time and dive time increased with lower dive-surface blow rate.

The impact variables accounting for variation in respiration responses were mainly associated with vessel distances as opposed to acoustic metrics. However, accumulated SEL from vessels was significant for several respiration variables. As vessels approached a gray whale, their respiration intervals significantly decreased with a higher surface blow rate and decreased dive time. With an increasing amount of accumulated SEL, whales spent less time at the surface with a decreased dive-surface blow rate. The distance of the closest seismic vessel also appeared to have altered the gray whales dive-surface blow rate, surface blow rate, and time at the surface (Table 2).

**Table 2:** Behavioral response model results for respiration variables relative to natural and impact explanatory variables. Numbers indicate coefficients and bold values represent significance (*P* < 0.05). “SV_Distance” and “Ves_Distance” refer to the closest seismic and other vessel, respectively, distance to the whale. “Ves_cSEL” is the accumulated SEL from vessels at each respiration bin

		Behavioral state (reference = feeding)							
Variables	*B*_0_	Feeding/travel	Traveling	Mixed	Depth	Tide height	SV_Distance	Ves_cSEL	Ves_Distance
Respiration interval	1.182	***0.020***	***0.166***	***0.047***	0.003				***0.042***
Surface time	0.812				***0.029***	0.118		*** − ***0.002	
Dive time	1.799	*** − 0.184***	*** − 0.859***	***-0.379***	***0.131***	0.315			0.223
Blows per surface time	2.399	*** − 0.042***	*** − 0.226***	***-0.476***	**0.091**				
Surface blow rate	6.716	*** − 0.340***	*** − 1.357***	***-0.523***	*** − 0.067***		0.105		***-0.941***
Dive-surface blow rate	1.500				*** − 0.018***	*** − ***0.081	*** − 0.016***	*** − 0.003***	
Time at surface	32.010	***1.614***	***6.255***	***3.627***	*** − 0.288***		*** − ***0.376	*** − 0.076***	
PC1	*** − ***0.930	***-0.188***	*** − 0.877***	*** − 0.328***	*** − ***0.032			***0.010***	
PC2	0.059	0.218	1.360	0.519	*** − 0.068***	*** − ***0.350			
PC3	3.157				*** − 0.150***	*** − ***0.324		*** − 0.009***	*** − 0.519***

Similar to the respiration models, behavioral state and water depth were the largest predictors for the movement response variables. Traveling gray whales exhibited higher speeds, longer ranges, and more directionality than feeding or feeding/traveling whales (Table 3). Gray whales were noted to have higher speeds and directionality in deeper water depths. Time of day was a predictor for a few response variables by BIC selection, but the coefficient was particularly small (< 0.00001) and *p* values not significant. Both seismic and vessel sounds and distances explained a significant amount of the variation in a number of response variables. As a seismic vessel approached a gray whale, the gray whale’s speed, range, and distance from shore increased. Whales also moved more perpendicular to vessels as vessels approached whales at closer distances. As seismic sound exposure levels increased, whales appeared to move closer to shore. With increasing continuous sound exposure levels from vessels, gray whales increased their reorientation rate and decreased their directionality. As a vessel approached closer to whales, whales altered their direction of movement. This was similar to the whale’s relative orientation to the vessel that was influenced by the proximity and direction the vessel approaching the whale. Principal component score responses reflected the results of the individual responses. Behavioral state was the largest predictor of the first two principal components. The remaining variation in the third component was significantly associated with water depth, vessel distance, and accumulated sound energy level.

**Table 3:** Behavioral response model results for movement variables relative to natural and impact variables. Numbers indicate coefficients and bold values represent significance (*P* < 0.05). “ROV_CV” represents the relative orientation to the closest vessel

			Behavioral State (reference = feeding)									
Variables	B_0_	Feeding/Travel		Traveling	Mixed	Depth	Time of Day	Seismic_SEL	SV_Distance	Ves_SEL	Ves_Distance	ROV_CV:Min_Range
Speed	0.765	***0.371***		***2.222***	***0.756***	***0.078***			***-0.074***			
Reorientation rate	29.666	*** − 10.603***		*** − 20.814***	*** − 6.234***	*** − 0.437***				0.041		
Linearity	0.657	***0.219***		***0.311***	***0.120***	***0.005***				*** − 0.001***		
Mean direction	0.591	***0.170***		***0.317***	***0.091***	***0.006***		0.001		*** − 0.001***		
Range	7.745	***7.756***		***38.650***	***11.708***	***1.217***			*** − 0.977***			
Distance from shore	0.084	***0.016***		***0.043***	***-0.002***	***0.161***		*** − 0.001***	*** − 0.034***			
cos(direction of movement)	0.645					0.014	0.000	0.038			*** − 0.013***	0.000
sin(direction of movement)	*** − ***0.075	0.006		0.105	0.070						***0.005***	
ROW_CV	*** − ***25.905								4.054		0.081	0.003
ROW_SV	*** − ***117.298						0.000		***15.152***		0.550	
PC1	0.873	*** − 1.511***		*** − 3.292***	*** − 1.116***	*** − 0.110***			***0.117***	***0.008***		
PC2	0.532	***0.254***		*** − 0.033***	***0.007***	*** − 0.041***			*** − 0.065***			
PC3	*** − ***0.278	*** − 0.464***		***0.094***	*** − 0.009***	***0.098***	***0.000***		*** − 0.111***			

#### Individual responses to activities

**Fig. 4:**
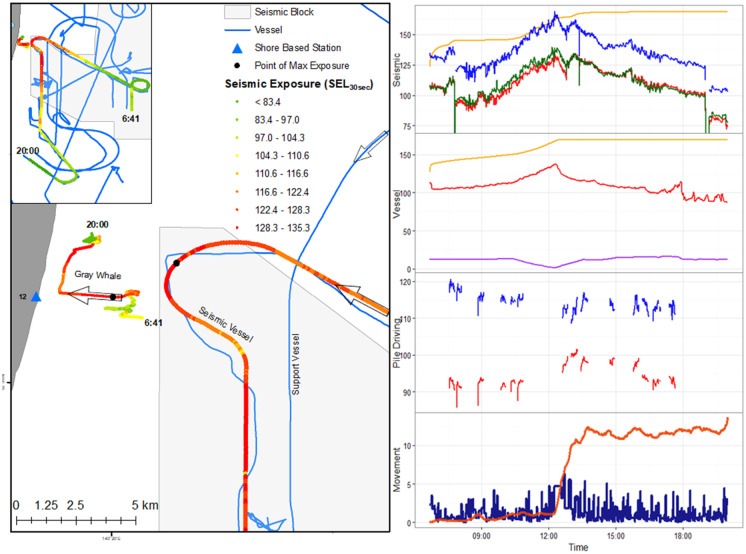
Individual gray whale movement (13.1-h track) while exposed to seismic, pile driving, and vessel sounds. The accumulated sound exposure level (cSEL; orange), 30-s sound exposure level (SEL_30s_; red), sound pressure level (SPL; green), peak sound pressure level (SPK; blue), closest vessel distance (purple), gray whale speed (km/h, dark blue), and gray whale displacement (the squared distance (km^2^) an animal moved from the point of origin, dark orange) are represented in the right graph for the timeline. The color scheme in the map of the seismic vessel and whale tracks represents the SEL_30s_ the whale received at that time, while the purple arrow in the map indicates the direction of vessel movement

Moderate to subtle changes in marine mammal behavior in response to anthropogenic activity are likely to be more frequent compared to larger responses. In addition, the responses can be context dependent on the activity that occurred at the time. However, examining obvious responses provides insight to developing an appropriate analytical framework for future analyses. As such, we provide a case example of a gray whale feeding as a seismic vessel approached (Fig. 4). The gray whale altered its feeding behavior to traveling towards shore as the seismic source vessel turned towards the whale’s direction. However, as the seismic vessel moved farther away from the whale, the whale resumed feeding albeit in an area different from the whale’s original feeding area.

## Discussion

### Sample size

The amount of seismic activity that occurred near western gray whales in 2015 provided a unique opportunity to advance our understanding of behavioral responses at the population level for western gray whales to anthropogenic activity. Gailey et al. (2016) illustrated the statistical sample sizes needed to detect moderate to subtle behavioral changes in western gray whale movements and respirations in relation to sound exposure. Relatively small sample sizes (< 60) were required to detect large (50% change in the response variable) changes in gray whale movements and respiration, while sample sizes of 300–1700 were required to detect subtle changes (10% change) among the response variables analyzed in this study. Previous behavioral response studies on western gray whales during seismic activity (Gailey et al., 2007, 2016) were limited in duration (3 weeks) and behavioral monitoring teams (1–2 teams). The five behavioral teams deployed in 2015 coupled with the duration of the surveys (4 months) yielded sample sizes four times greater than all the previous combined behavioral response studies on western gray whales to seismic activity. As such, this study had a higher probability to detect more moderate to subtle (< 50%) changes in movement and respiration variables and in fact documented clear evidence of responses to industrial sounds.

### Prey availability

Due to the extent of the feeding area, detailed documentation of the benthic communities is challenging, which in turn presents challenges to understanding how the benthic community changes within the gray whales foraging grounds. Point samples were taken from certain grid areas during three separate periods (Blanchard et al., 2022a, b). To estimate biomass values where whales were observed to be feeding, we attempted both kriging and IDW interpolations. It is arguable that the more complex kriging approach provided an advantage over the IDW approach from an estimation perspective, but was limited in scope by the narrow data requirements for kriging. Gray whales were observed to be feeding in areas with higher Amphipoda biomass, a preferential prey (Blanchard et al., 2019; Demchenko, 2010; Demchenko et al., 2016), compared to traveling or other behavioral states observed.

Although prey availability has been previously suggested as an explanatory variable within behavioral response models, this study found that prey biomass does not particularly alter gray whale movement or respiration patterns. This suggests gray whale movement and respirations patterns while engaged in feeding or traveling activities were similar regardless of prey biomass. It is possible that prey availability would alter the gray whale’s foraging strategy (feeding/travel versus feeding), spatial distribution, and abundance rather than the gray whale’s behavioral movement or respiration behaviors.

### Other natural influences on gray whale behavior

Natural spatial and temporal variables were found to have little association with gray whale behavior, suggesting that western gray whales move and respire similarly throughout the nearshore feeding habitat and their foraging season. The spatial variable “Station” was not found to explain the variability in any of the response variables and time of day entered into a few models, but the coefficients were particularly small and found to be inconclusive as explanatory variables. Interestingly, water depth was one of the largest predictors of gray whale movement and respiration patterns, as noted in previous studies, and was particularly important for dive time (Gailey, 2013; Gailey et al., 2007, 2016). Studies on eastern gray whales have also reported changes in dive time, surface time, and surface-blow rate associated with increasing water depth (Guerrero, 1989; Mallonée, 1991; Stelle et al., 2008; Würsig et al., 1986). However, this study had not only a strong association with water depth for respiration, but also for gray whale movement patterns. This could be partially related to sample sizes, anthropogenic activity, or a different foraging strategy. Gailey et al. (2022) reported that gray whales in 2015 were unusually far from shore in the northern part of the study area during the middle part of the feeding season. Blanchard et al. (2022a, b) also observed a seasonal high abundance of sand lance in that area, which could indicate that the gray whales observed farther from shore were feeding on a seasonal more mobile prey, which could have influenced the response variables analyzed.

The behavioral state of gray whales significantly influenced the majority of their movement and respiration patterns. Gray whales feeding in a centralized location had slower speeds and more variable directionality compared to traveling whales. Gray whales also increase their amount of time below the surface while feeding compared to traveling. It was unsurprising, however, that the dive-surface blow rate was not significantly associated with behavioral state since this variable is more or less a physiological constant. However, Gailey et al. (2016) found few associations with behavioral state in a number of the respiration models that conducted similar analyses, albeit with a drastically reduced sample size (36 focal follows, 395 bins). Behavioral states were classified based primarily on observations of the animal’s movement and respiration behaviors while on the surface; one could expect, therefore, the vast majority of the variation to be explained by these categorical definitions. However, despite inclusion of behavioral states, significant variation remained in the models.

### Sound and vessel influence on gray whale behavior

Vessel distances and sound exposure significantly changed gray whale movement and respiration patterns. Whales were breathing faster and moving at higher speeds when vessels were closer to whales or when sound exposures were higher. Similar to Gailey et al. (2016), the gray whale direction of movement was significantly related to the direction and approach distance of the closest vessel. The association between the relative orientation of the whale and vessel was intended to examine impacts from a context-based approach (Ellison et al., 2012). Whales would likely behave differently if the source was moving toward or away from the whale. Gray whales could also respond differently depending on the location of the source relative to the whale’s current direction. For example, despite receiving similar sound exposures, migrating gray whales notably responded to a sound source directly in their migration route, but little behavioral response was observed when the same source was placed outside of their migratory pathway (Tyack & Clark, 1998). Gailey et al. (2016) and the case study illustrated here present similar context-based responses. Whales farther from shore were more likely to be closer to the vessels. The relationship of gray whale distance from shore and vessel proximity indicated that whales were moving closer to shore to increase their distance from the vessels.

Chronic exposure to disrupting activities, even with low levels of exposure, can lead to displacement out of critically important foraging habitats. With an increasing amount of anthropogenic activity, there could be a potential for increased tolerance or habituation to sound and/or vessel activity. Alternatively, gray whales could become sensitized to anthropogenic activity, especially following previous negative experiences. It is unknown in this study how frequently a single individual or group of individuals (e.g., pregnant females) repeatedly responded to anthropogenic activities. Schwarz et al. (2022) demonstrate that a portion of the population remained in the offshore feeding area and younger gray whales, which could be more naïve to previous exposures, are more likely to occur in the nearshore feeding area. It is possible that some individuals who are sensitized to anthropogenic activity could be over-represented in the analyses. However, concurrent shore-based photo-identification with tracking suggests that greater than 60% of the individuals identified by all five photo-identification teams were represented to some degree within the analyses.

Repetitive disruption of gray whale foraging activities accumulates over time and could ultimately result in biologically significant impacts to individuals and thus the population. For example, bioenergetic studies estimated that a loss of 10 feeding days or 3% reduction of energy intake due to disturbance could lead to an unsuccessful pregnancy (McHuron et al., 2021; Villegas-Amtmann et al., 2015, 2017). Other studies have also hypothesized that industrial activities off Sakhalin could be one contributing factor towards the observed slow recovery of this endangered population (Weller et al., 2002a). Simulation studies further suggested that calf survival could be impacted in the future when pregnant females arrived on the foraging grounds in poorer body condition during a year of disturbance activity (McHuron et al., 2021). Although gray whales have been documented to abandon entire lagoons for years and up to a decade as a result of anthropogenic activity (Bryant et al., 1984; Jones et al., 1994), western gray whales have a high site fidelity and annual return to the Sakhalin foraging area despite the increasing amount of human related activity occurring on their foraging habitat. This could suggest that they may have no alternative or comparable foraging habitat other than the foraging areas off Sakhalin Island. While eastern gray whales have been shown to forage over larger ranges (Lagerquist et al., 2019), the western gray whale known foraging range is relatively small (Mate et al., 2015; Muir et al., 2015a, b), which may make this population more susceptible to anthropogenic or environmental perturbations (Gailey et al., 2020; Lowry et al., 2018).

## Conclusion

The results of this study found multiple indicators of behavioral response to seismic and vessel sounds and proximity. These results are similar to those found in a study associated with a seismic survey conducted in 2001 in the same study area (Gailey et al., 2007; Weller et al., 2002b; Yazvenko et al., 2007) but differ from those found in a study associated with a seismic survey in 2010 (Gailey et al., 2016; Muir et al., 2015a, b, 2016), for which Gailey et al. (2016) concluded that samples sizes had insufficient statistical power to detect moderate to subtle changes in behavior. This suggests that anthropogenic activities off Sakhalin Island impacted gray whale foraging activities, at least in the short-term. Although gray whales responded on a population level to the activities, it is unknown if the responses resulted in biologically significant (i.e., decreased growth, survival, reproductive success) impacts to individuals or the population. Mitigation measures applied during 2015 could have reduced larger/long-term responses and sensitization to activities, but this study found that it did not eliminate all behavioral responses of gray whales relative to the anthropogenic activities that occurred off Sakhalin Island. Future studies should investigate the longevity of behavioral response in western gray whales to the activities as well as examine responses within components of the population (e.g., mother-calf pairs, pregnant females, immature adults) to better understand the energetic loss as a result of the activities. In addition, individual heterogeneity and repetitive responses should be further explored relative to natural covariates and anthropogenic exposure as a substantial amount of variability existed in the movement and respiration datasets that required large sample sizes to identify responses. Some of the components of the population, such as pregnant females and mother/calves, could be more sensitive to anthropogenic activity but their sensitivity was not directly examined in this study due to the inability to identify all individuals from shore. A behavioral dose–response model (Dunlop et al., 2018) should also be developed to re-examine the 163 dB re 1 μPa^2^ SPL criteria that have historically been used as a mitigation measure to reduce disruption to gray whale feeding activities. Disruption to feeding activities as a result of seismic and vessels on their foraging ground should also not be viewed in isolation. For example, (Gailey et al., 2020) found a correlation between the number of foraging days (as determined by sea-ice conditions off Sakhalin) and reproductive success and calf survival. Of particular concern are the few reproductive females in this small population that have significantly higher energetic costs due to their pregnancy and calf rearing (Christiansen et al., 2018; Villegas-Amtmann et al., 2015, 2017). Local fisheries and the possibility of entanglement could also result in further disturbance and survival of individuals within this population (Lowry et al., 2018). As gray whales have a number of other factors impacting their future survival throughout their home range, it is important to continue to monitor and examine the effectiveness of mitigation strategies employed off Sakhalin to rapidly assess potential impacts and address these management issues in the future.
